# Inhibition of Rumen Methanogens by a Novel Archaeal Lytic Enzyme Displayed on Tailored Bionanoparticles

**DOI:** 10.3389/fmicb.2018.02378

**Published:** 2018-10-09

**Authors:** Eric Altermann, Linley R. Schofield, Ron S. Ronimus, Amy K. Beattie, Kerri Reilly

**Affiliations:** ^1^Rumen Microbiology, Animal Science, AgResearch Ltd., Palmerston North, New Zealand; ^2^Riddet Institute, Massey University, Palmerston North, New Zealand; ^3^Soil Biology, Forage Science, AgResearch Ltd., Christchurch, New Zealand

**Keywords:** bionanoparticles, methane mitigation, lytic enzyme, archaea, methanogens, PHA, polyhydroxyalcanoate, rumen

## Abstract

Methane is a potent greenhouse gas, 25 times more efficient at trapping heat than carbon dioxide. Ruminant methane emissions contribute almost 30% to anthropogenic sources of global atmospheric methane levels and a reduction in methane emissions would significantly contribute to slowing global temperature rises. Here we demonstrate the use of a lytic enyzme, PeiR, from a methanogen virus that infects *Methanobrevibacter ruminantium* M1 as an effective agent inhibiting a range of rumen methanogen strains in pure culture. We determined the substrate specificity of soluble PeiR and demonstrated that the enzyme is capable of hydrolysing the pseudomurein cell walls of methanogens. Subsequently, *peiR* was fused to the polyhydroxyalkanoate (PHA) synthase gene *phaC* and displayed on the surface of PHA bionanoparticles (BNPs) expressed in *Eschericia coli via* one-step biosynthesis. These tailored BNPs were capable of lysing not only the original methanogen host strain, but a wide range of other rumen methanogen strains *in vitro.* Methane production was reduced by up to 97% for 5 days post-inoculation in the *in vitro* assay. We propose that tailored BNPs carrying anti-methanogen enzymes represent a new class of methane inhibitors. Tailored BNPs can be rapidly developed and may be able to modulate the methanogen community *in vivo* with the aim to lower ruminant methane emissions without impacting animal productivity.

## Introduction

Agriculture contributes between 3 and 4% to the global gross domestic product (GDP)^1^. A growing world population dictates an increasing demand on food suppliers and both crop and livestock production indices have been steadily rising. Such an intensification in production comes at the cost of a growing carbon footprint. Agricultural emissions are a recognized contribution to anthropological climate change and emissions continue to grow annually (Tubiello et al., 2015). In particular, meat, wool, and dairy production relies on ruminants which annually produce ∼80,000,000 metric tons of methane, contributing almost 30% of global methane emissions^2^.

Methane in ruminants is produced predominantly in the forestomach, commonly known as the rumen, as part of ruminal feed by microbes which creates nutrients and metabolites for the ruminants. One of the major by-products of this fermentation process is hydrogen and a specialized group of archaea, known as methanogens, remove free hydrogen through the reduction of carbon dioxide to methane. Previous research has shown that methanogens can be inhibited in the rumen using chlorinated inhibitors such as bromochloromethane, where a 30% decrease in methane production was observed (Denman et al., 2007; Bagi et al., 2017). However, the use of such compounds is prohibited in food production and other avenues must be found to effectively reduce ruminant methane emissions. More recently, the non-chlorinated inhibitor 3-nitrooxypropanol (3-NOP) has been tested in cows where it successfully reduced methane emissions (Hristov et al., 2015). 3-NOP is metabolized by methanogens and the inhibited methyl-coenzyme M reductase (MCR) enzyme activity is restored over time (Hristov et al., 2015; Duin et al., 2016). A potentially limiting factor of 3-NOP is the varying susceptibility of different methanogen groups with as much as a 100-fold variation in growth inhibition (Ragsdale, 2016). More detailed information on 3-NOP and other small molecule inhibitors can be found in the comprehensive review article published by Patra et al. (2017). Changing feed and feed composition has also been shown to reduce methane emissions. For example, exclusively feeding rape over 15 weeks resulted in a reduction in methane emissions by up to 30% in sheep, whereas feeding chicory or white clover lead to inconsistent results when compared to diets based on rye grass (Sun et al., 2015). However, rape may comprise a much higher nitrogen and sulfur content than rye grass, possibly leading to elevated nitrous oxide emissions of the ruminants through fecal matter, depending on seasonal variations and growth conditions. Alternative feed additives, such as tannins, oils or fats may reduce methane emissions by up to 18 – 20% but must be carefully monitored to avoid negative health effects due to sudden introduction or over ingestion and availability may be limited or seasonal (Liu et al., 2011; Kolling et al., 2018; Wang et al., 2018). Another promising feed additive is the seaweed *Asparagopsis* that has been shown to reduce methane emissions by up to 80% (Machado et al., 2014). However, the active compound bromoform, is a brominated organic solvent and known animal carcinogen. In all cases, changing animal diets or adding supplements needs to be carefully assessed with regards to seasonal availability, total emission balances and possible toxicity to the animals and humans. For a comprehensive review on nutritional strategies in ruminants and their impacts on methane emissions, please refer to these review articles (Buddle et al., 2011; Hristov et al., 2013; Hatew, 2015; McGrath et al., 2018).

Alternative methane reduction strategies require effective, cost-efficient and non-toxic (environmentally friendly) mechanisms that specifically target rumen methanogen cells without negatively impacting on the microbial plant fiber degradation or on animal production parameters.

One such strategy, phage therapy, has been used in biomedical applications since 1920 (McAuliffe et al., 2007). However, phage therapy using intact virions is host-strain specific and the production of virus tail-like structures or even purified enzymes is costly and complex. Further, the cell wall makeup of some methanogenic archaea, in particular within the order Methanobacteriales, differs significantly to the peptidoglycan of bacteria (Kandler and König, 1993) (**Supplementary Figure 5**). Pseudomurein, the major compound of the cell wall of members of Methanobacteriales is composed of *N*-acetyl-*L*-talosaminuronic acid and *N*-acetyl-D-glucosamine connected through β(1–3) glycosidic linkages. These chemical differences make pseudomurein inert to known bacterial cell wall hydrolases and to lysozyme (Kandler and König, 1993; Visweswaran et al., 2010).

To date, only a few methanogen viruses have been described in some detail (Luo et al., 2001, 2002; Krupovic and Bamford, 2008; Chien et al., 2013; Weidenbach et al., 2017) (for an excellent review on archaeal viruses in general refer to Krupovic (Krupovic et al., 2018), while more insights are emerging through the analysis of rumen (viral) metagenomes (Berg Miller et al., 2012; Ross et al., 2013; Yutin et al., 2015; Anderson et al., 2017). Similarly, only two methanogen phage lytic enzymes have been described in detail previously; PeiP of the *Methanothermobacter marburgensis* phage ψM2 (Pfister et al., 1998) and PeiW of the *Methanothermobacter wolfeii* phage ψM100 (Luo et al., 2001). Both are capable of hydrolysing the ε-(alanine)-lysine bond of the peptide linker between layers of pseudomurein (Luo et al., 2002). Sequence analyses carried out for PeiP and PeiW highlighted that both enzymes contain four N-terminal pseudomurein binding repeat (PMBR) domains, essential for cell wall binding (Visweswaran et al., 2010), and a C-terminal catalytic domain (Visweswaran et al., 2010), which is in opposite orientation to the structural makeup of lysins of Gram-positive bacteria. Both PeiP and PeiW share a significant level of sequence similarity to each other (50% identity, 62% positives) with individual domains being particularly highly conserved. Genome sequencing the rumen methanogen *Methanobrevibacter ruminantium* M1, a third methanogen lysin was discovered, PeiR of the *Methanobrevibacterium* prophage φmru (Leahy et al., 2010). To our knowledge, only a single rumen methanogen provirus has been described in detail, *Methanobrevibacter ruminantium* M1 virus Φmru and evidence was provided that the lytic enzyme PeiR was active against the host strain in pure culture (Leahy et al., 2010).

A novel delivery and enzyme production system in the form of polyhydroxyalkanoate (PHA) bionanoparticles (BNPs) has been developed (Peters and Rehm, 2005, 2006). A number of microbes are capable of synthesizing PHA as carbon and energy reservoirs (Keshavarz and Roy, 2010). The key enzyme for PHA biosynthesis is the PHA synthase PhaC (Yuan et al., 2001) which forms the polyester by linking *(R)*-3-hydroxyacyl-CoA thioester. The resulting biopolymer remains covalently bound to PhaC. The hydrophobic polyester strands aggregate into spherical BNPs with the strands in the core and attached proteins forming the surface. BNPs have proven to be effective, cost-efficient (Choi and Lee, 1997) and non-toxic in previous studies and can make up to 80–90% of the cell’s dry weight (Philip et al., 2007). These structures currently receive significant biotechnological interest in a wide range of applications, including agriculture (Keshavarz and Roy, 2010). Polyhydroxyalkanoate (PHA), the main component of one type of BNPs produced by recombinant cell factories, has gained FDA approval as a food additive (Philip et al., 2007). It has also been shown that the key enzyme, PhaC, tolerates protein fusions to both its C- and N-termini and the resulting tailored BNPs have the unique ability to display proteins and enzymes on their surface in an oriented fashion while retaining their respective native enzyme activities. For a comprehensive review on tailored BNPs and their applications refer to Rehm (Rehm, 2010).

In the proof-of-concept work presented here we investigated the predicted domain structure of the lytic enzyme PeiR from *Methanobrevibacter ruminantium* M1 and determined the enzyme characterisitcs of the free enzyme to understand its relation to other, known archaeal lytic enzymes, and its substrate specificity. To evaluate the potential of tailored BNPs active against rumen methanogen strains *in vitro*, we created fusion proteins and displayed PeiR on the surface of BNPs. We demonstrated that PeiR-tailored BNPs were active against a wide range of rumen methanogen strains in pure culture and provided effective growth and methane inhibition for several days. Combining phage therapy with the accessibility and scalability of tailored BNP production has resulted in a conceptually new approach to mitigate ruminant methane emissions that may also be used in many other applications.

## Materials and Methods

### Microbial Strains and Culture Conditions

Bacterial strains and their respective sources, plasmids, and oligonucleotides used in this study are listed in **Table 1**. *Methanobrevibacter ruminantium* M1 (DSM 1093), *Methanobrevibacter* sp. AbM4, *Methanobrevibacter* sp. 229/11, *Methanobrevibacter* sp. D5, *Methanosarcina barkeri* CM1, *Methanosphaera* sp. A4, and *Methanobacterium formicicum* BRM9 were grown anaerobically from frozen stocks in Hungate tubes in 10 ml of medium RM02 as described elsewhere (Kenters et al., 2011). Filter-sterilized vitamin 10 solution (0.1 ml per 10 ml culture tube) (Joblin et al., 1990), supplemented with rumen fluid (final concentration 25%), methanol (20 mM), sodium formate (60 mM), sodium acetate (20 mM) and coenzyme M (CoM) (10 μM) were added to the medium after autoclaving (Leahy et al., 2010; Wedlock et al., 2010). For growth assays, growing methanogen cultures were anaerobically subcultured into fresh supplemented RM02 medium and incubated at 39°C without agitation in Hungate tubes (Wedlock et al., 2010). The headspace was pumped with high pressure hydrogen to 1.4 bar to facilitate methanogen growth. Cell suspensions for both cell suspension and agarose plate lysate assays were prepared as follows; cells were harvested (12,000 × *g*, 15 min, 4°C), washed with 50 mM MOPS-NaOH pH 7.0 containing 1 mM DTT, 7.3 mM K_2_HPO_4_ and 5% glycerol (v/v) and resuspended at 50 mg ml^-1^ in the same buffer before storing at -20°C until further use. *Ruminococcus flavefaciens* FD-1 was grown anaerobically from frozen stock in BY medium (Joblin, 2005) supplemented with 4% (w/v) cellulose at 39°C.

**Table 1:** Microbial strains, plasmids and amplification oligonucleotides.

	Relevant characteristics	Source or reference
**Microbial Strains**		
*Escherichia coli*		
XL1 Blue	endA1 gyrA96(nal*^R^*) thi-1 recA1 relA1 lac glnV44 F’[ ::Tn10 proAB^+^ lacI^q^ Δ(lacZ)M15] hsdR17(r_K_^-^ m_K_^+^) pMCS69	Stratagene
BL21 λ(DE3)	F^-^ ompT gal dcm lon hsdS_B_(r_B_^-^ m_B_^-^) λ(DE3 [lacI lacUV5-T7 gene 1 ind1 sam7 nin5])	Novagene
BL21-Rosetta 2 (DE3)	F^-^ *ompT hsdS*_B_(r_B_^-^ m_B_^-^) *gal dcm* (DE3) pRARE2 (Cam*^R^*)	Novagene
*Methanobrevibacter ruminantium* M1	DSM1093, ATCC 35063, type strain Bovine rumen isolate	(Leahy et al., 2010) DSMZ^(a)^
*Methanobrevibacter ruminantium* 31A	New Zealand deer rumen isolate	PGgRc*^(b),^* NZAGRC^(c)^, AgResearch Ltd.
*Methanobrevibacter* sp. AbM4	New Zealand ovine abomasum isolate	(Leahy et al., 2013) PGgRc^(b),^ NZAGRC^(c)^, AgResearch Ltd.
*Methanobrevibacter* sp. 229/11	New Zealand ovine rumen isolate	(Kelly et al., 2016a) PGgRc^(b),^ NZAGRC^(c)^, AgResearch Ltd.
*Methanobrevibacter* sp. D5	New Zealand ovine rumen isolate	PGgRc^(b),^ NZAGRC^(c)^, AgResearch Ltd.
*Methanobrevibacter smithii* DSM 861	ATCC 35061, type strain Sewer digester isolate	DSMZ^(a)^ (Smith, 1966)
*Methanobrevibacter gottschalkii* DSM 11977	Type strain Horse feces	(Miller et al., 1986) DSMZ^(a)^
*Methanobrevibacter* sp. SM9	New Zealand sheep rumen isolate	(Kelly et al., 2016b) PGgRc^(b),^ NZAGRC^(c)^, AgResearch Ltd.
*Methanothermobacter thermautotrophicus* (ΔH)	DSM1053, ATCC 29096, type strain Sewage sludge isolate	DSMZ^(a)^ (Zeikus and Wolee, 1972)
*Methanobacterium formicicum* BRM9	New Zealand bovine rumen isolate	(Kelly et al., 2014) PGgRc^(b),^ NZAGRC^(c)^, AgResearch Ltd.
*Methanosphaera stadtmanae* DSM 3091	ATCC 43021, type strain Human feces isolate	DSMZ^(a)^ (Miller and Wolin, 1983)
*Methanosphaera* sp. A4	New Zealand Wallaby fore stomach isolate	PGgRc^(b),^ NZAGRC^(c)^, AgResearch Ltd.
*Methanospirillum hungatei* DSM 864	ATCC 27890, type strain Sewage sludge isolate	NZMS^(d)^ (Ferry et al., 1974)
*Methanosarcina barkeri* CM1	New Zealand bovine rumen isolate	(Lambie et al., 2015) PGgRc^(b),^ NZAGRC^(c)^, AgResearch Ltd.
*Ruminococcus flavefaciens* FD-1	Rumen bacterium capable of degrading the cellulose, hemicellulose and pectin present in plant cell walls	University of Illinois at Urbana-Champaign, United States
**Plasmids**		
pCR2.1-TOPO	P_lac_ LacZα F1_ori_ Kan^R^ Amp^R^ pUC_ori_	Invitrogen Life Technologies
pMCS69	pBBR1MCS [Cmr, mob+, tra-, lacPOZ’ (Kovach et al., 1995)] containing the phbA-phbB genes downstream of lac promoter	Amara and Rehm, 2003
pET151D TOPO	pBR322 origin, Amp^R^, P_T7_, LacI, TOPO cloning site, 6xHis	Invitrogen Life Technologies
pETC	pET-14b derivative coding for the phaC wild type under T7 promoter control	Peters and Rehm, 2008
pET14b	Ap^R^; T7-promotor, phaC-linker-malE	Novagen, Madison, WI, United States
pKRpeiR-C	Ap^R^; T7-promotor, phaC-linker-peiR	This study
		
Oligonucleotides		
PeiR-Fwd	5′-CTCGAGATGGTTCGTTTTAGCCGTGATATGC-3’	This study
PeiR-Rev	5′-GGATCCTTATGCCGGACACACAACATAATAATTCTGG-3′	This study
PeiRfree-Fwd	5′-CACCATGGTTAGATTCAGCAGAGAC-3′	This study
PeiRfree-Rev	5′-TCATGCAGGACAGACAACATAGTAG-3′	This study


*^*(a)*^DSMZ, Deutsche Sammlung von Mikroorganismen und Zellkulturen;^*(b)*^PGgRc, Pastoral Greenhouse Gas Research Consortium; ^*(c)*^NZAGRC, New Zealand Agricultural Greenhouse Gas Research Centre; ^*(d)*^NZMS, New Zealand Microbiological Society.*

### Bioinformatic and Phylogenetic Analyses

The genome sequence of the integrated *M. ruminantium* M1 provirus φmru was annotated through the GAMOLA2 software package (Altermann et al., 2017). Original Pfam (database release version 27) and TIGRfam (database release version 13.0) results for the open reading frame associated with the lytic enzyme PeiR were investigated individually. Newly identified PeiR pseudomurein binding repeat (PMBR) domains were added to the GAMOLA2 annotation after defining respective motif boundaries by cross-referencing Pfam domain alignments. C39-peptidase (*n* = 37) and PMBR Pfam domain sequences (*n* = 109) were retrieved from the NCBI online amino acid database via their respective accession numbers using custom software and NCBI’s E-utilities^3^. Amino acid sequences of individual PeiR PMBR and C39-peptidase domains were aligned and adjusted to their respective Pfam seed sequences in Geneious Pro version 5.6.2.^4^ Full-length amino acid sequences of the lytic enzymes PeiR, PeiP and PeiW were aligned using ClustalX 2.1.^5^ Sequence alignments of individual domains were generated with RaxML (Stamatakis, 2006) (Maximum Likelihood with 100 rapid bootstraps) for larger datasets and with MEGA5^6^ for phylogenetic reconstructions. Evolutionary relations of the amino acid sequences of the PMBR domains of PeiR, PeiP, and PeiW were inferred by the Maximum Likelihood method and visualized using MEGA5.

### Cloning, Expression and Purification of PeiR-Free Enzyme

Primers were designed using the annotated gene sequence of *peiR* (Leahy et al., 2010; Schofield et al., 2015). PCR primers (PeiRfree-Fwd and PeiRfree-Rev) were used and an N-terminal 6 residue histidine affinity purification tag and a peptidase (rTEV) cleavage site were added (**Table 1**). The PCR and subsequent transformation of the *Escherichia coli* expression strain BL21-Rosetta 2 (DE3) (Novagen, United States) were as described previously (Schofield et al., 2015).

PeiR was expressed in *E. coli* and purified using nickel affinity chromatography as described earlier (Schofield et al., 2015), with the following modifications. LB medium (10 g*^-l^* Peptone, 5 g*^-l^* yeast extract, 10 g*^-l^* NaCl) was used for 800 ml growth and induction was initiated with 0.5 mM isopropyl-β-D-1-thiogalactopyranoside (IPTG) when the cell culture reached a density of 0.50 at 600 nm, monitored by measuring optical densities of samples in cuvettes with a 1 cm path length. Cultures were then incubated for approximately 6 h. Cell pellets were thawed and resuspended in 3 volumes lysis buffer [50 mM Tris pH 7.5 containing 2 mM DTT, 300 mM NaCl, 10 mM imidazole, 1% Triton X-100 (v/v), 20% glycerol (v/v)] without the addition of EDTA-free protease inhibitor (Roche, Auckland, New Zealand). The filtered enzyme was applied (1.5 ml min^-1^) to a HIS-Select^TM^ Nickel-affinity gel (Sigma-Aldrich, New Zealand) column (2.5 × 18 cm) equilibrated with 20 mM Tris pH 7.5 containing 2 mM DTT, 0.3 M NaCl, 10 mM imidazole, 1% Triton X-100 (v/v), 20% glycerol (v/v), 5 mM CaCl_2_ and 10 mM MgCl_2_. The imidazole was removed, and buffer exchanged, using a Bio-Gel^®^ P-6DG (Bio-Rad, United States) column (2.5 × 48 cm). Electrophoresis in the presence of SDS and protein concentration determinations were performed following the methods of Schofield et al. (2015).

### Enzymatic Assays of Free PeiR Enzyme

The native molecular mass for PeiR was determined using gel filtration chromatography as described previously (Schofield et al., 2015). Spectrophotometric measurements were performed and pH values reported as described previously (Schofield et al., 2015).

Agarose plate lysate assays were carried out as described previously (Schofield et al., 2015) to detect activity of free PeiR enzyme on dead methanogen cells incorporated into agarose plates. Samples of 28 μl of PeiR (0.2 mg) or control buffer were used.

Cell suspension assay reaction mixtures contained 125 μl dead M1 cells [up to 50 mg ml^-1^ in 50 mM MOPS-NaOH pH 7.0 containing 1 mM DTT, 7.3 mM K_2_HPO_4_ and 5% glycerol (v/v)], 10 mM TCEP and 10 mM MgCl_2_ in 50 mM Bistris propane [1,3-bis(tris(hydroxymethyl)methylamino)propane] buffer at pH 8.0 and were preincubated at 40°C for 7–10 min. The dilution of the cell suspension in the assay reaction mixture gave an initial OD_600_ of approximately 1.0. The reaction was initiated by the addition of 100 μl of PeiR (0.22 mg). The total volume of the assay was 850 μl. A decrease in optical density of an M1 cell suspension was monitored over time, aerobically, at 600 nm (Bush, 1985). The contents of cuvettes were mixed thoroughly, immediately before each measurement to resuspend cells. Between measurements, cuvettes were maintained at 40°C in a thermostatted water bath.

Synthetic peptide plate assays were performed as described previously (Schofield et al., 2015). Activity was detected as a zone of yellow color in the plate due to release of pNA from the synthetic peptide substrates. Samples of 40 μl of PeiR (0.12 mg) or control buffer were used.

For liquid assays, synthetic peptides L-Ala-pNA (ApNA), L-Glu-γ-pNA (EγpNA), H-Glu-Ala-pNA (EApNA), Glu-γ-Ala-pNA (EγApNA), Glu-γ-Ser-pNA (EγSpNA), Glu-γ-Thr-pNA (EγTpNA) and Asp-β-Ala-pNA (DβApNA) were obtained, solutions prepared and assays performed as described in Schofield et al. (2015). The standard aerobic assay reaction mixture contained 45 μl (100 μg) PeiR, 10 mM DTT and 10% glycerol (v/v) in 50 mM Bistris propane buffer at pH 7.0 and was preincubated at 40°C for 7–10 min. The reaction was initiated by the addition of 40 μl prewarmed peptide to give a final concentration of 2.1 mM. The total volume was 130 μl. The metal salts tested in assays were MgCl_2_⋅6H_2_O (BDH, England) and CaCl_2_⋅2H_2_O (Biolab, Australia) and were dissolved in water which had been pretreated with Chelex 100 resin (Bio-Rad, Hercules, CA, United States). The final concentration of divalent metal salt was 10 mM in the assay reaction mixture.

### Plasmid Construction for PhaC and PhaC-PeiR BNP Production

The *peiR* DNA sequence was codon optimized for expression in *E. coli* and synthesized by GeneArt (Regensburg, Germany). The sequence was subsequently amplified from the synthesized construct using oligonucleotides containing 5′-and 3′ -restriction sites. The 5′ -primer, PeiR-Fwd, contained an XhoI restriction site, while the 3′ -primer, PeiR-Rev, harbored a BamHI site (**Table 1**). DNA was amplified using Platinum PCR SuperMix High Fidelity (Life Technologies Auckland, New Zealand). PCRs were carried out as follows: 94°C for 3 min (initial denaturation step), followed by 10 cycles of 94°C for 15 s, 65°C for 30 s and 72°C for 40 s, dropping 2°C every 2 cycles until a final annealing temperature of 55°C was reached, followed by 30 cycles of 94°C for 15 s, 55°C for 30 s 72°C for 40 s (amplification) and a final extension step of 72°C for 10 min. Cycling was carried out on a Mastercycler S thermocycler (Eppendorf, Hauppauge, NY, United States). The resulting PCR amplicons were TA-cloned into the plasmid pCR2.1 according to the manufacturer’s instructions (Life Technologies). Resulting plasmids were sequenced (Massey Genome Service, Institute of Fundamental Science, Massey University, New Zealand^7^) and plasmids with verified inserts digested with XhoI and BamHI to excise the *peiR* DNA fragment. The *peiR* DNA fragment was purified from a 0.8% (w/v) agarose gel run in TAE buffer at 100 V using a Min-elute gel purification column (Qiagen, Hilden, Germany) and ligated into the pET14b plasmid digested with XhoI and BamHI (**Supplementary Figure 1B**). The resulting fusion plasmid pKRpeiR-C (**Table 1**) was transformed into *E. coli* XL1 Blue cells (Stratagene, La Jolla, CA, United States) for screening. The sequence validated pKRpeiR-C plasmid was then transformed into *E. coli* BL21 λ(DE3) competent cells, which harbored the helper plasmid pMCS69 (Amara and Rehm, 2003). pMCS69 is a pBBR1MCS derivative and features the β-ketothiolase (phbA) and acetoacetyl-CoA reductase (phbB) genes from *Ralstonia eutropha* that yield (*R*)-3-hydroxybutyryl-CoA from acetyl-CoA. PhaC control beads were produced by the plasmid pETC (**Table 1**), which encodes the wild-type PhaC. pETC was transformed into *E. coli* BL21 λ(DE3) competent cells containing the plasmid pMCS69.

### Production and Purification of PhaC and PhaC-PeiR BNPs

*Eschericia coli* BL21 λ(DE3) containing the pKRpeiR-C or pETC plasmids were grown aerobically in Luria-Bertani (LB) broth supplemented with ampicillin (50 μg ml^-1^), chloramphenicol (64 μg ml^-1^) and 1% (w/v) glucose at 37°C. 2 l flanged Erlenmeyer flasks were filled with 1 l LB broth and *E. coli* was incubated with shaking (rpm = 150). Oxygen was allowed to diffuse into the flasks through loosely fitted aluminum covers. When the OD_600_ reached 0.5, BNP production was induced with 1 mM IPTG followed by agitation at 25°C for 48 h. PHA BNPs displaying PhaC-PeiR or PhaC (control) on their surface were spun down (6000 *g*, 5 min at 4°C), resuspended in 50 mM phosphate buffer pH 7.5 and lysed via sonication in a Vibracell sonicator (Sonics and Materials, Newtown, CT, United States) on ice at power level 2 at 40% duty for 20 s bursts over a 10 min time period with 30 s rest intervals. PHA BNPs were purified by ultracentrifugation over a glycerol gradient as previously described (Brandl et al., 1988). Briefly, lysed cells were added to a 2-step glycerol gradient of 44% (v/v) glycerol in 50 mM phosphate buffer pH 7.5 layered on 88% (v/v) glycerol in 50 mM phosphate buffer and centrifuged at 21,000 × *g* for 2 h at 4°C in a Sorvall TH641 swing-out rotor (Thermofisher Scientific, Auckland, New Zealand) in a Sorvall RC100 ultracentrifuge. BNPs were recovered from the gradient interface by pipetting.

PhaC and PhaC-PeiR BNPs were resuspended in 20 mM MOPS pH 7.0 containing 1 mM DTT, 0.3 M NaCl and 20% glycerol (v/v) and stored at -80°C. PHA BNPs were visualized with Nile Red staining described previously (Peters and Rehm, 2008) and fluorescence microscopy using a Leica DM2500 microscope (Leica, Wetzlar, Germany) with UV filter (excitation BP 355–425 nm).

### PHA BNP Protein Analyses

Protein profiles of PHA BNPs were analyzed by SDS-PAGE on 4–15% pre-cast gradient polyacrylamide gels (Bio-Rad, Hercules, CA, United States). Gels were stained with Safestain (Life Technologies Auckland, New Zealand) and protein bands recorded using a Nikon D700 dSLR camera custom-mounted to a Kodak Gel Logic 200 Imaging System. Protein sizes were determined by comparison to the Precision plus dual color protein marker 10–250 kD range (Bio-Rad, United States). To confirm the PhaC-PeiR protein fusion and full-length expression of both proteins, a protein band close to the predicted molecular mass of the PhaC-PeiR fusion protein was excised from the acrylamide gel and trypsin digested. The subsequent peptide mix was analyzed by LC-MS/MS mass spectrometry on an amaZon speed ETD (Bruker Corporation, Fremont, CA, United States) and resulting data was searched and aligned to known protein sequences present in the non-redundant NCBI database using the Mascot Search Engine (version 2.4.0, Matrix Science, London, United Kingdom) and ProteinScape version 3.0.^8^

### Transmission Electron Microscopy

PhaC-PeiR BNPs were fixed in 3% (v/v) glutaraldehyde, 2% (v/v) formaldehyde in 0.1 M K_2_HPO_4_ buffer pH 7.2 for 2 h at room temperature before washing, dehydration and embedding in resin. Sections (1 μm in thickness) were cut from trimmed resin blocks using a glass knife and an EM UC7 Ultramicrotome (Leica, Wetzlar, Germany). Sections were heat-mounted onto a glass slide and stained with 0.05 % (w/v) toluidine blue. Examination of the specimens was conducted using a Philips CM 10 transmission electron microscope (Philips, Amsterdam, Netherlands) and documented with a Morada digital camera (Olympus SIS, Muenster, Germany).

### Methanogen Growth Studies and Methane Measurements

Growing methanogen cultures were transferred into fresh RM02 medium in Hungate tubes using a 1% (v/v) inoculum. The culture headspace was then replaced with a H2/CO2 (80:20 v/v) mixture to remove any trace of methane present. PhaC (negative control) or PhaC-PeiR (treatment) BNPs (200 μl, wet weight 10 mg) were added and cultures incubated as described above. Cell growth was monitored every 24 h for up to 5 days by measuring OD_600_ using a Spectronic 200 (Thermofisher Scientific, Auckland, New Zealand), directly inserting the tubes into the spectrophotometer.

Evaluating changes in optical density required consideration of two additional parameters: (a) the addition of BNPs increased the optical density and (b) PhaC-PeiR BNPs formed larger aggregates over time, thus changing background light absorbance. To account for those changes, optical densities were corrected according to Equation 1.

$${OD}_{600\left(n\right)}={OD}_{600\left(n\right)\left[a\right]}-({OD}_{600\left(0)[bc\right]}-{OD}_{600\left(0)[M1c\right]})+\left({OD}_{600\left(n-1)[bead\right]}-{OD}_{600\left(n\right)[bead]}\right)$$

Equation 1: Calculation of corrected optical density values. OD_600_: optical density measured at a wavelength of 600 nm; n: sample taken at predefined time point; OD_600(*n*)_: corrected optical density at point n; OD_600(*n*)[α]_: measured optical density at point n; OD_600(0)_: measured optical density at time 0; OD_600(*n*-1)_: measured optical density at point n-1; bc: PhaC-PeiR treatment or PhaC control B plus cells; M1c: *M. ruminantium* M1 (control A) plus cells; bead: PhaC or PhaC-PeiR beads in the absence of methanogen cells. Briefly, the increase in optical density due to the addition of BNPs was subtracted from measured optical densities. Additionally, changes in optical densities of BNPs in the absence of cells over time were calculated and also allowed for in the final optical density.

Statistically significant differences between treatment groups were determined using linear regression analyses for independent biological repetitions with internal replicates and One Way ANOVA analyses for individual experiments with internal replication (SigmaPlot 12.5, Systat Software Inc., United States)^9^.

The amount of methane present in the headspace was measured at 24 h intervals by gas chromatography using a Varian Aerograph 660 instrument (Walnut Creek, CA, United States) and calculated according to Equation 2. Immediately after gas samples were taken, the headspace was flushed with hydrogen to reset the methane partial pressures and to provide sufficient levels of hydrogen for methanogen growth. All experiments were performed in biological triplicates.

$${CH}_{4}[ml]=\frac{\frac{{Peak.Height}_{\left(sample\right)}}{{Vol.loaded}_{\left(sample\right)}}\times \frac{{Attenuation}_{\left(Sample\right)}}{{Attenuation}_{\left(S\,\tan dard\right)}}\times Vess.Gas.Vol}{\frac{{Avg.Peak.Height}_{(S\,\tan dard}}{{Vol.loaded}_{\left(S\,\tan dard\right)}}\times \frac{100}{{Concentration}_{\left(S\,\tan dard\right)}}}$$

Equation 2: Calculation of the amount of methane (CH_4_) in a gas sample. Samples were taken from Hungate tubes using gas tight syringes where the pressure in the syringe was equal to the pressure in the Hungate tube. The gas pressure in the syringes was not released before injection into the detector. Peak.Height: height of the corresponding CH_4_ peak in the gas chromatography analysis; Vol.loaded: Gas volume loaded into the detector [ml]; Avg.Peak.Height: Average Peak Height of a known standard; Vess.Gas.Vol: Vessel Gas Volume [ml]; Concentration: concentration of standard [%].

## Results

### The Lytic Enzyme of the Methanogen Virus φmru, PeiR

**FIGURE 1:**
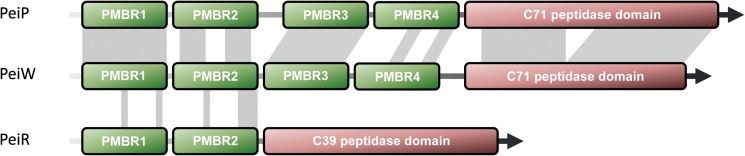
Modular structure of three methanogen lytic enzymes. Green boxes indicate identified pseudomurein binding repeat (PMBR) domains, red boxes depict the C71 and C39 peptidase domains. Gray shading shows conserved amino acids. PeiP, pseudomurein endoisopeptidase of *Methanothermobacter marburgensis* phage ψM2; PeiW, pseudomurein endoisopeptidase of *Methanothermobacter wolfeii* phage ψM100; PeiR, endopeptidase of integrated *M. ruminantium* M1 provirus φmru. Not drawn to scale.

The lytic enzyme PeiR is markedly different in both amino acid sequence and modular composition from PeiP and PeiW. While PeiP and PeiW are similar in length, PeiR is much shorter, sharing only 32 and 44% of its sequence with PeiW and PeiP, respectively. Yet, PeiR has a similar structural makeup to PeiP and PeiW, featuring a block of N-terminal pseudomurein binding repeat (PMBR) domains and a C-terminal catalytic domain (**Figure 1**).

Both PeiP and PeiW feature 4 PMBR domains that share extensive sequence similarity, while PeiR harbors only 2 PMBR domains. The PMBR Pfam family currently includes 124 sequences from 26 species, grouped into 28 architectures. PMBR domains are not only found in lytic enzymes, but also in a range of other cell-wall binding proteins. The PeiR PMBR domains, however, share very little sequence similarity to PeiP, PeiW or any other known PMBR domains. A Pfam search still detected both PeiR PMBR domains, but at a level well above the combined inclusion threshold (*E*-value: 0.11, score 12.4). An unrooted phylogenetic Maximum Likelihood analysis using PFAM PMBR seed sequences highlighted not only the uniqueness of each PeiR PMBR domain to other known domains, but also to each other (**Supplementary Figure 2**) and a more focused Maximum Likelihood analysis including only the PeiR, PeiP and PeiW PMBR domains further strengthened this observation (**Supplementary Figure 3**).

The catalytic domains of both PeiP and PeiW belong to the Peptidase_C71 PFAM family and are similar to other thiol proteases and transglutaminases (Visweswaran et al., 2010). PeiR on the other hand has a catalytic domain belonging to the CA clan of the Peptidase_C39 PFAM family. This family also includes a range of peptide bacteriocin maturation proteases and many families of viral endopeptidases (Barrett and Rawlings, 2001). A TIGRfam hit (TIGR01193) further supports this classification and describes ABC-type bacteriocin transporters whose N-terminal domain processes the amino terminal leader peptide of bacteriocins. Both types of peptidases are cysteine endopeptidases, requiring reducing conditions for biological activity. Similar to the PeiR PMBR domains, the PeiR peptidase domain is only distantly related to other known members of the C39 family (**Supplementary Figure 4**).

### Specificity and Activity of the Free Recombinant PeiR Enzyme Against Synthetic Substrates and Methanogen Strains

Recombinant His-tagged PeiR protein was purified to homogeneity in one step using nickel-affinity chromatography with a final concentration of 0.68 mg PeiR ml^-1^. PeiR was active in both cell suspension and synthetic peptide assays under aerobic conditions in the presence of a reducing agent such dithiothreitol (DTT) or tris (2-carboxyethyl) phosphine (TCEP) (results not shown). PeiR activity was not dependent on the presence or absence of divalent metal ions.

A variety of synthetic peptide substrates were tested in an agarose plate activity assay to obtain an indication of substrate specificity. This type of assay gives the highest chance of detecting activity as the plate is incubated both anaerobically and for an extended period. The synthetic peptides mimic the bonds within the peptide chain of pseudomurein (**Supplementary Figure 5**). PeiR showed some activity on glutamate-γ-threonine-p-nitroaniline (EγTpNA), but no activity on the other substrates tested (**Supplementary Table 1**). This activity is consistent with the structure of the peptide link in the pseudomurein of *M. ruminantium* M1, which is reported to contain threonine in place of alanine (Kandler and König, 1993; König et al., 1994). In comparison, PeiW and PeiP show activity on EγApNA (Schofield et al., 2015) and lyse the ε-isopeptide bond between alanine and lysine in the peptide chain (Luo et al., 2002).

A further means of determining the substrate specificity of recombinant PeiR used a variety of dead but intact methanogen cells in agarose plate lysate assays (**Supplementary Figure 6**). PeiR lysed cells from *Methanobrevibacter ruminantium* M1 (20 mm diameter of clear zone, SD = ± 0.8 mm, *n* = 3) and *Methanothermobacter thermautotrophicus* ΔH (19 mm diameter of clear zone, SD = ± 0.8 mm, *n* = 3). Other methanogens strains [*Methanobacterium formicicum* BRM9, *Methanobrevibacter gottschalkii* (DSM 11977), *Methanobrevibacter ruminantium* 31A, *Methanobrevibacter smithii* (DSM 861), *Methanobrevibacter* sp. SM9, *Methanosarcina barkeri* CM1, *Methanosphaera stadtmanae* (DSM 3091), and *Methanospirillum hungatei* (DSM 864)] were only qualitatively tested for PeiR mediated cell lysis (without repetition), to establish a frame of reference for PhaC-PeiR BNPs. A zone of clearing was observed with sometimes an inner zone of whitening. Methanobacteriales all possess pseudomurein, with the peptide link usually containing alanine, threonine or serine at the cleavage site (Kandler and König, 1993; König et al., 1994). PeiR was very poor at lysing *Methanospirillum hungatei* and CM1, which do not contain pseudomurein in their cell wall; rather they contain glycoprotein and methanochondroitin, respectively (Kandler and König, 1993; Albers and Meyer, 2011). The low but definitive activity against the latter two strains remains mechanistically unresolved and may reflect non-specific proteolysis.

### Specificity and Activity of PhaC-PeiR BNPs Against *M. ruminantium* M1

**FIGURE 2:**
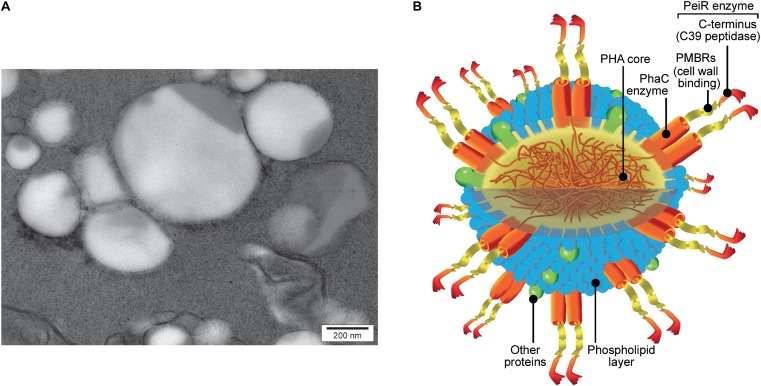
PhaC-PeiR tailored BNPs. **(A)** Transmission electron micrograph of purified PhaC-PeiR BNPs. Beads assume a roughly spherical shape and vary in average size between 50 and 500 nm. **(B)** Artists’ rendition of a BNP. The polyhydroxyalkanoate (PHA) hydrophobic core is encompassed by a phospholipid layer in which the PhaC enzyme is embedded as a dimer. Each PhaC monomer carries a PeiR protein fusion that mediates pseudomurein binding through two pseudomurein binding repeat (PMBR) domains and proteolytic activity through the C-terminal C39 peptidase domain. Individual features are not drawn to scale.

A C-terminal PhaC fusion was created combining the PhaC synthase and the PeiR lytic enzyme, placing the catalytic C39 endopeptidase domain at the C-terminal end of the fusion protein. Subsequent expression of the *phaC-peiR* fusion gene in *E. coli* resulted in PHA BNPs and the presence of the lytic enzyme PeiR on those BNPs was confirmed via mass spectrometry (**Figure 2** and **Supplementary Figures 1A–C**).

**FIGURE 3:**
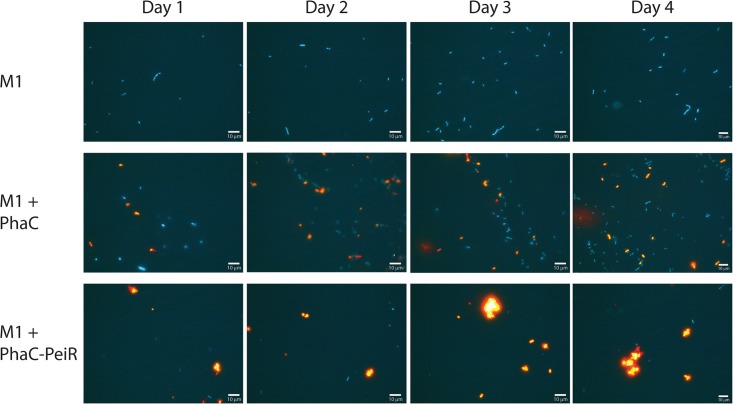
Fluorescence micrographs of M1 cells, and M1 cells with added PhaC BNPs (M1 + PhaC) and with added PhaC-PeiR BNPs (M1 + PhaC-PeiR). Samples were taken over 4 days post-BNP addition and representative images selected. M1 cells are shown in blue and Nile Red-loaded PhaC and PhaC-PeiR BNPs appear in orange.

The effect of PhaC-PeiR BNPs on the growth of M1 was qualitatively monitored over time using fluorescence microscopy. Over the course of 4 days, M1 cells and those supplemented with control PhaC BNPs were metabolically active as exhibited by blue-green autofluorescence, typical for viable methanogens (**Figure 3**, ‘M1’ and ‘M1 + PhaC’). Cell numbers steadily increased over time, indicating that the culture was also capable of growth. In contrast, exposure of M1 to PhaC-PeiR tailored BNPs reduced cell numbers immediately (**Figure 3**, ‘M1 + PhaC-PeiR’). Over time, the number of visible cells remained almost undetectable, indicating that new methanogen cells continued to be susceptible to PhaC-PeiR BNPs. Interestingly, PhaC-PeiR BNPs consistently exhibited a trend to form larger aggregates over time. This aggregation was not observed for PhaC control beads and it is likely that the presence of PeiR PMBR domains may mediate such an aggregation.

**FIGURE 4:**
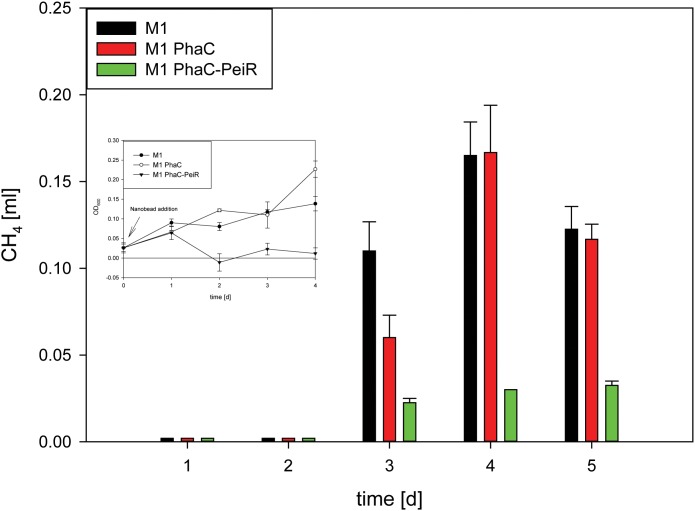
Methane production in pure cultures of M1. To avoid build-up of methane in the culture medium prior to BNP addition, beads were added at the time of culture inoculation (*t* = 0 d) ‘M1’: *M. ruminantium* M1 without BNPs (control A), ‘M1 PhaC’: *M. ruminantium* M1 with PhaC BNPs added (control B), ‘M1 PhaC-PeiR’: *M. ruminantium* M1 with PhaC-PeiR BNPs added. Inset: Corresponding growth assay. Error bars represent the standard error from 4 repetitions.

The lytic activity of PhaC-PeiR BNPs was quantified by measuring changes in optical density (OD) of cultures growing in liquid media (**Figure 4**). M1 was incubated for 2 days prior to BNP addition to test for positive cell growth. Untreated M1 cells and those supplemented with PhaC BNPs entered the exponential growth phase after 3 days and stationary phase after 6 days post inoculation, although PhaC-supplemented cell cultures consistently reached higher optical densities (*n* = 32). The addition of PhaC-PeiR BNPs resulted in a distinct drop in optical density for up to 3 days post-BNP addition, after which cell growth resumed slowly. A one-way ANOVA test carried out using 4 independent experiments (*n* = 16) confirmed that PhaC-PeiR BNPs significantly inhibited OD increases in cultures of M1 (*P*-value ≤ 0.05) for all days post-BNP addition.

While cell lysis is a good indirect indicator for a reduced methane production, methane production was measurement directly on pure cultures of M1 (**Figure 4**). The addition of PhaC-PeiR BNPs to a growing culture of M1 significantly reduced methane levels by up to 81.2% for 5 days compared to untreated and PhaC-supplemented cultures, mirrored by the lack of cell growth measured by optical density (**Figure 4**, inset) (*P* < 0.05).

### Specificity and Activity of PhaC-PeiR BNPs Against Other Rumen Methanogen Strains

Six pure cultures of methanogen strains isolated from the rumen environment (**Table 1**) were exposed to PhaC-PeiR BNPs to assess the potential impact of a C-terminally BNP-immobilized PeiR on target range and biological activity on live methanogens.

**FIGURE 5:**
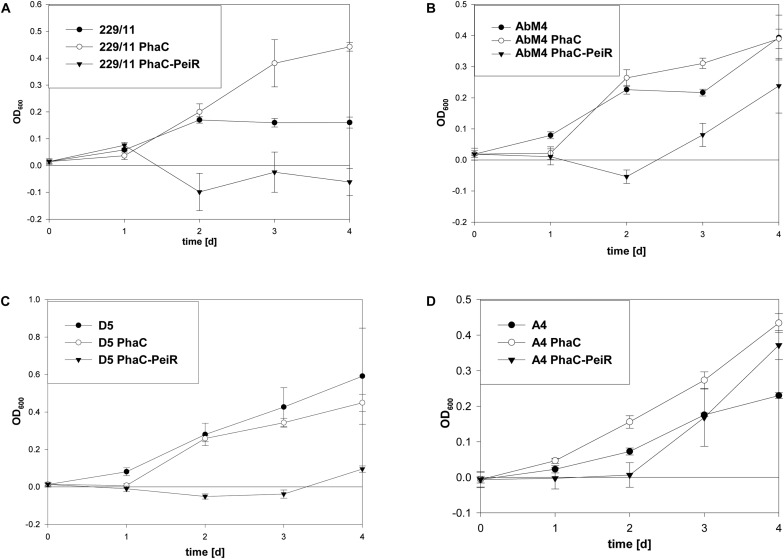
Changes in optical density (OD) by PhaC-PeiR tailored BNPs against rumen methanogens. Graphs show the respective growth inhibition assays indicated as a drop in optical density measured at 600 nm. **(A)** 229/11, **(B)** AbM4, **(C)** D5, **(D)** A4. Error bars represent the standard error from 4 repetitions. BNPs were added at time *t* = 0 d.

**FIGURE 6:**
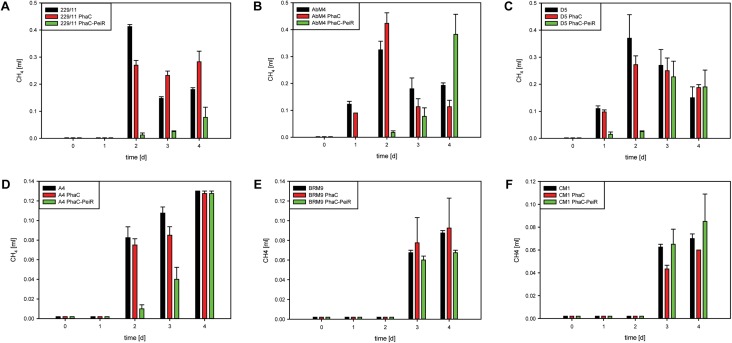
Biological activity of PhaC-PeiR tailored BNPs against rumen methanogens. Graphs depict methane production in pure cultures. **(A)** 229/11, **(B)** AbM4, **(C)** D5, **(D)** A4, **(E)** BRM9, **(F)** CM1. Error bars represent the standard error from 4 repetitions. To avoid build-up of methane in the culture medium prior to BNP addition, beads were added at the time of culture inoculation (*t* = 0 d). For CM1 and BRM9, methane was measured as a cumulative value over time due to low production. All other samples had their respective headspace flushed after sampling to remove any residual methane. BNPs were added at time *t* = 0 d for 229/11, AbM4, D5 and A4, and 2 days prior to methanogen cell inoculation for CM1 and BRM9 to allow for extended anoxic calibration.

229/11, AbM4, and D5 all exhibited significant inhibition of growth and methane production (**Figures 5**, **6A–C**, respectively) for up to 72 h post-bead addition (Repeated Measures ANOVA, *p* ≤ 0.05). Interestingly, the degree and duration of PhaC-PeiR mediated inhibition decreased with increasing phylogenetic distance of the respective methanogen strain to the original PeiR host M1. To further investigate this trend, three rumen methanogen strains outside the genus *Methanobrevibacter* clade were tested for their respective response to PhaC-PeiR BNPs. Both A4 and BRM9 were inhibited by PhaC-PeiR BNPs at reduced levels compared to *Methanobrevibacter* ssp. A4 showed the strongest response (**Figures 5**, **6D**, respectively) with inhibition detectable up to 3 days post-inoculation, while methane production by BRM9 was significantly inhibited only on day 3 (**Figure 6E**), although good inhibition was seen in the agarose plate lysate assay. Surprisingly, the addition of PhaC and PhaC-PeiR BNPs to a growing culture of BRM9 resulted in the formation of large, macroscopic aggregates of BNPs (**Supplementary Figure 7**), a phenomenon not observed with any other rumen methanogen strain investigated. The third methanogen strain outside the genus *Methanobrevibacter* tested, CM1, has a cell wall made up predominantly of galactosamine, carbohydrates and uronic acids (Albers and Meyer, 2011). Exposure to PhaC-PeiR BNPs did not result in a statistically significant reduction in methane production over the entire length of the *in vitro* assays (Repeated Measures ANOVA, *p* > 0.05, **Figure 6F**). These results support the hypothesis that PeiR is able to recognize a wide range of pseudomurein substrates, but cannot hydrolyse other types of cell envelopes. A detailed analysis of the responses of individual methanogen strains to PhaC and PhaC-PeiR BNPs can be found in **Supplementary Text 1**.

## Discussion

The first lytic enzyme tested as an effective anti-methanogen agent was PeiR from the *M. ruminantium* M1 integrated provirus φmru. The overall enzyme architecture of PeiP, PeiW and PeiR is similar, but the detailed makeup does differ significantly. It has been previously shown that a minimum of three PMBR domains were required for the surface (S)-layer protein MTH719 of *Methanothermobacter thermautotrophicus* to bind to the pseudomurein cell wall (Visweswaran et al., 2011). Interestingly, the presence of only two PMBR domains in the active PeiR enzyme may point to a more effective binding mechanism than previously known. Compared to the known methanogen lytic enzymes PeiW and PeiP, we propose that PeiR represents a new type of archaeal virus endopeptidase. The presence of only two novel PMBR domains, in combination with a clan CA protease, is sufficient for effective cell wall binding and subsequent cell lysis of the host strain M1 (**Figure 3**).

Despite the difference in peptidase domains, the biological activity of PeiR appears similar to that reported for PeiW and PeiP which cleave the ε-isopeptide bond between alanine and lysine (Visweswaran et al., 2010). In M1, the alanine is replaced by threonine in the pseudomurein peptide side chain and PeiR is able to hydrolyse a synthetic substrate that mimics the glutamate-threonine peptide bond. No activity was detected on other substrates where threonine is replaced. The free PeiR enzyme was active against a much wider range of methanogens than implied by the synthetic substrates. It is noteworthy that PeiR is active against cell walls of *Methanothermobacter thermautotrophicus* – which is sensitive to PeiW and PeiP - at the same level as tested *Methanobrevibacter* spp. PeiR was also active against *Methanosphaera stadtmanae* which contains serine in place of alanine in the pseudomurein cell wall (Biavati et al., 1988), while the corresponding synthetic substrate was not recognized. In contrast to PeiW and PeiP, PeiR does not require divalent metal ions for activity and is not inhibited when treated with EDTA (Luo et al., 2002; Schofield et al., 2015). At present, the seemingly wide range of biological activity of PeiR free enzyme cannot be mechanistically explained and further structural and biochemical studies are required to unravel the possible recognition sites and enzymatic mechanisms on native methanogen cell substrates.

Industrial production of rumen methanogen inhibitors ideally comprises a simple *in situ* production process that does not require any complex and expensive post-processing procedures. Microbial cells produce tailored PHA BNPs displaying functional proteins in a one-step process that does not require any physical or biochemical modification beyond a basic cell disruption to free the BNPs (Draper and Rehm, 2012). This implies cost effective large-scale production of individual BNP-types and offers an attractive system for initial *in vitro* models and subsequent upscale production. The enzyme that polymerises *(R)*-3-hydroxyacyl-CoA thioester monomers into polyester, PHA synthase PhaC, tolerates both N- and C-terminal fusions (Grage et al., 2009) and a C-terminal fusion between PhaC and PeiR was shown to be biologically active.

The effectiveness of a BNP-based anti-methanogen product may also be influenced by the range of sensitive rumen methanogens. Both the free lytic enzyme PeiR and the enzyme immobilized on PHA BNPs exhibited a remarkable versatility against a wide range of different methanogen strains. Effective inhibition and reduction in methane was achieved for *Methanobrevibacter* spp., *Methanobacterium formicicum* and a *Methanosphaera* sp. All four *Methanobrevibacter* spp. tested were sensitive to PhaC-PeiR BNPs, albeit with a graduated response. This decrease in sensitivity continued for strains A4 and BRM9. A published phylogenetic framework for rumen methanogens (Jeyanathan et al., 2011) inferred relationships between type strains of the methanogen clades investigated here. The lytic enzyme PeiR was identified in M1 and provided the most potent effect on its host strain. Selecting strain M1 as reference point in the inferred phylogenetic tree, the level and duration of PhaC-PeiR inhibition correlated well with the respective phylogenetic distance, making a compelling argument for an increasingly different cell wall makeup that even within the same genus contributes to varying phenotypes.

Interestingly, the presence of cells of BRM9 led to aggregate formation of PhaC and PhaC-PeiR BNPs. These aggregates resembled the rod-like cell morphology of BRM9, suggesting that BNPs may attach to an extracellular matrix produced by BRM9 cells. Veiga et al. (1997) reported that *Mb. formicicum* is able to produce extracellular polymers (ECP) composed of polysaccharides and polypeptides that play an important function in granule formation and cell-to-cell adhesion. The production of ECP by rumen methanogens has not been reported previously and it is tempting to speculate that such an interaction may provide an additional mechanism of methanogen inhibition by interference with ECP-mediated cell-to-cell adhesion or communication between methanogens and/or other syntrophic bacteria.

The use of lytic enzymes as antimicrobial agents has been described in other fields and is a particularly promising alternative to the use of antibiotics. A comprehensive assay on the development of the lysin CF-301, active against *Staphylococcus aureus* (MRSA), toward a novel therapeutic class has been recently published by Fischetti (2018). While lysins and related enzymes may successfully inhibit rumen methanogens today, they still face the same problem of emerging microbial resistance. However, their modular makeup enables a unique approach by creating chimeric enzymes with different modes of action, thereby bypassing emerging resistances (Manoharadas et al., 2009; Fernandes et al., 2012; Mao et al., 2013). In fact, libraries of lytic enzymes and their chimera could be established and displayed on BNPs, ready to replace current variations when resistance is detected. The short development cycle for creating new lytic-enzyme displaying BNP variants represents one of the major advantages of this technology and makes it much more flexible and future proof than other approaches that rely on a single compound and/or require much longer development cycles.

PhaC-PeiR BNPs offer effective methanogen inhibition for up to 5 days post-bead addition in pure culture. The average turnover of the rumen is between 7 h for liquids (Evans, 1981) and 14 h for solid particles (Owens et al., 1979). By inhibiting methanogens beyond this retention time, it is likely that the methanogen population may be washed out of the rumen, creating a longer lasting methane inhibition effect than can be measured in a static system. In combination with a continuous dose delivery system, recurrence of rumen methanogens may be prevented for extended periods of time.

While the tailored BNP platform holds much promise, there are also some known bottlenecks that need to be addressed before the technology can be applied in large scale and on-farm. The current two-plasmid system requires the application of two different antibiotics and is limiting scalability to fed-batch fermentations. Similarly, the current production host *E. coli* may not be the optimal vehicle for large scale fermentation processes, due to its requirements for oxygenation. Because the bacterial host is a genetically modified organism (GMO), application on-farm will require the removal of host cell material through separation processes.

## Conclusion

In this initial proof-of-concept *in vitro* work, we have shown that the lytic enzyme of a methanogen integrated provirus, PeiR, is biologically active both as a free enzyme and immobilized on the surface of PHA BNPs. PhaC-PeiR tailored BNPs were capable of inhibiting an exceptionally broad range of different rumen methanogen strains in pure culture, while significantly reducing methane production for several days (**Supplementary Text 2**). The current limitation of this study, using pure cultures, will be overcome in the future by validating PeiR-BNPs in increasingly complex rumen simulations, such as batch and fed-batch rumen fermenters, to deliver convincing evidence of efficacy and to optimize BNP manufacture processes before moving to animal experiments. Once suitable anti-methanogen enzymes have been selected, the development cycle of new anti-methanogen BNPs can be measured in weeks. This is of particular importance in engineering the next generation of tailored beads that may feature multiple proteins and enzymes that inhibit or specifically bind to rumen methanogens. The one-step biological synthesis of tailored beads and the economical purification are attractive protocols for future commercialization.

## Author Contributions

KR, AB, RR, and LS carried out the experimental work. EA carried out the computational analyses. EA and RR supervised the research. EA, KR, RR, and LS wrote the manuscript.

## Conflict of Interest Statement

EA, RR, and LS are listed as inventors on a patent protecting the lytic enyme PeiR and filed by the Pastoral Greenhouse Gas Research Consortium (PGgRc), New Zealand (Application number 12/678,936). The BNP technology platform is commercialized in New Zealand by PolyBatics, Ltd. The remaining authors declare that the research was conducted in the absence of any commercial or financial relationships that could be construed as a potential conflict of interest.

## Footnotes

1 http://data.worldbank.org

2 http://www.epa.gov

3 http://www.ncbi.nlm.nih.gov/books/NBK25501/

4 www.geneious.com

5 http://www.clustal.org/clustal2/

6 http://www.megasoftware.net/

7 http://genome.massey.ac.nz

8 www.bruker.com

9 http://www.sigmaplot.com/
