# Erratum: Inhibition of Rumen Methanogens by a Novel Archaeal Lytic Enzyme Displayed on Tailored Bionanoparticles

**DOI:** 10.3389/fmicb.2018.02982

**Published:** 2018-11-22

**Authors:** Frontiers Production Office

**Affiliations:** Frontiers Media SA, Switzerland

**Keywords:** bionanoparticles, methane mitigation, lytic enzyme, archaea, methanogens, PHA, polyhydroxyalcanoate, rumen

Due to a production error, the last name of the fourth author was incorrectly spelled as Amy K. Beatty. The correct form is Amy K. Beattie. The publisher apologizes for this mistake. The original article has been updated.

