# Supplementary material for: Inhibition of Rumen Methanogens by a Novel Archaeal Lytic Enzyme Displayed on Tailored Bionanoparticles
Source: Front Microbiol. 2018 Oct 9;9:2378. doi: 10.3389/fmicb.2018.02378 (PMC6189367; doi:10.3389/fmicb.2018.02378)
Supplement: Supplementary file 1 [file Data_Sheet_1.docx]

**Supplemental Tables**

Supplemental Table 1: Substrate specificity; activity of PeiR free enzyme on synthetic peptide substrates using an agarose plate assay. The presence of a zone of yellow colouration was compared; ‘–‘ no zone, ‘+’ observable zone. Ala: alanine; Glu: glutamate; Ser; serine; Thr: threonine; Asp: aspartate; pNA: *p*-nitroaniline

| Synthetic peptide substrate | Abbreviation | Relative activity |
| --- | --- | --- |
| L-Ala-pNA | ApNA | ₋ |
| H-Glu-Ala-pNA | EApNA | ₋ |
| Glu-γ-Ala-pNA (Glu(Ala-pNA)-OH) | EγApNA | ₋ |
| Glu-γ-Ser-pNA (Glu(Ser-pNA)-OH) | EγSpNA | ₋ |
| Glu-γ-Thr-pNA (Glu(Thr-pNA)-OH) | EγTpNA | ₊ |
| Asp-β-Ala-pNA (Asp(Ala-pNA)-OH) | DβApNA | ₋ |

**Supplemental Figures**


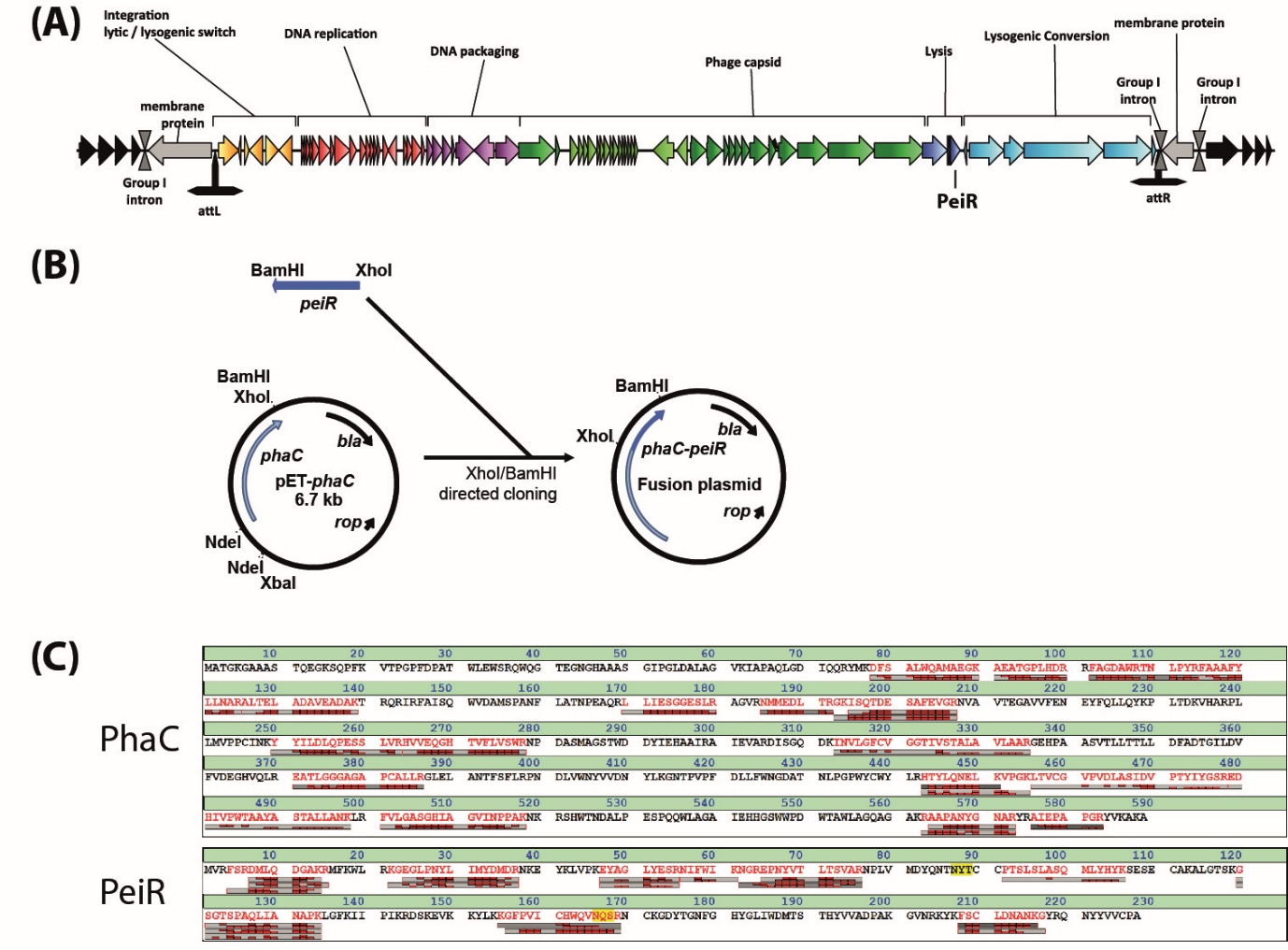


Supplemental Figure 1: Identification, cloning, and validation of PhaC-PeiR BNPs. (A) Schematic representation of the M1 integrated provirus genome of ϕmru. Coding regions are shown as arrows, with predicted functional modules colour coded. Attachment sites are shown as inverted ‘T’ (*attL* and *attR*). Individual open reading frames are drawn to scale. (B) Cloning scheme for C-terminal in-frame fusion of PeiR to PhaC using site-directed cloning. Gene fusion partner *peiR* is shown in blue, the plasmid bound PHA synthase, *phaC*, is depicted in light blue. The resulting gene fusion plasmid was sequence verified. (C) Mass spectrometry of PhaC-PeiR BNPs. Identified peptide fragments (in red with respective fragments in grey/red bars) were aligned against the deduced amino acid sequences of PeiR and PhaC.


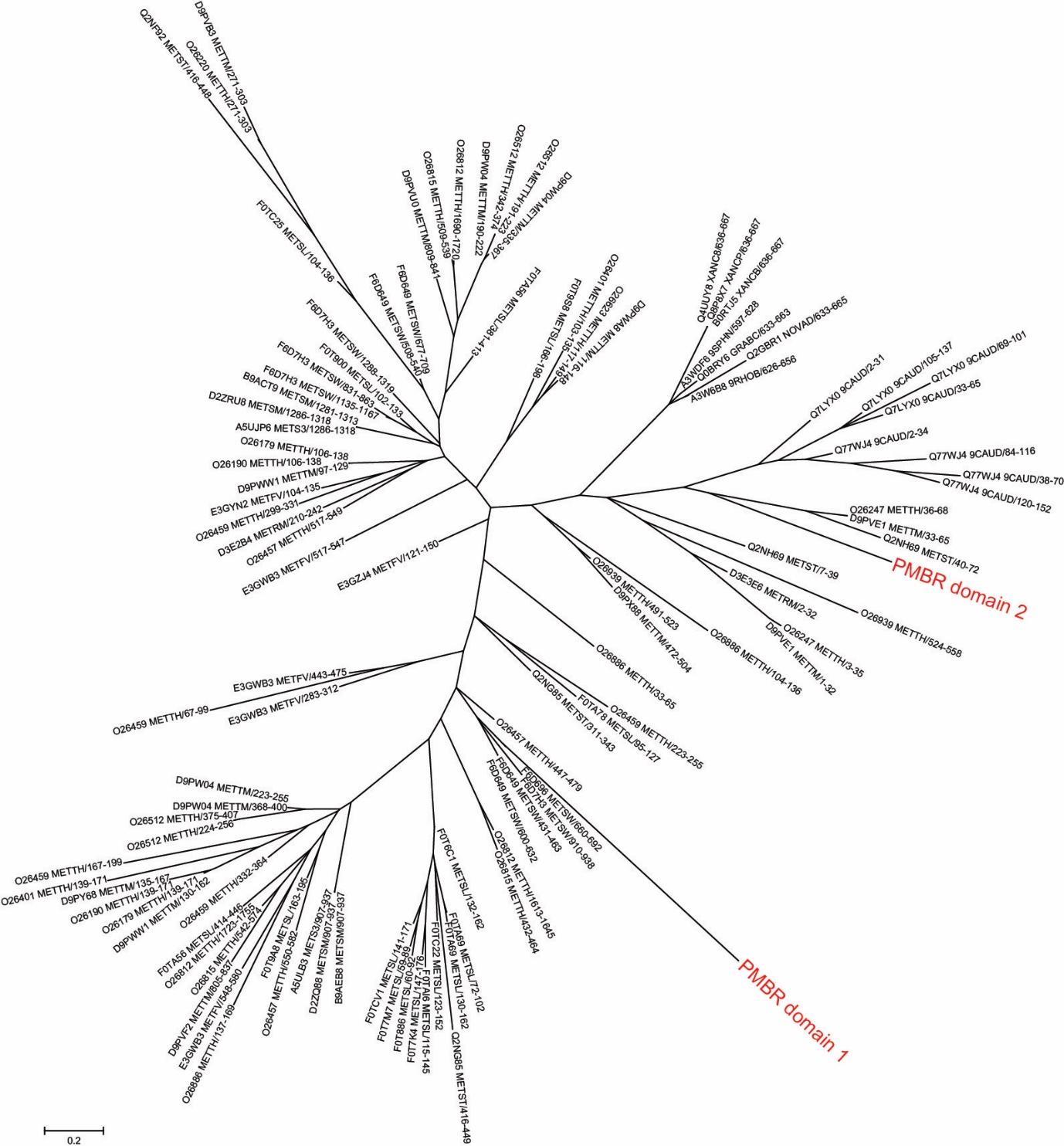


Supplemental Figure 2: Phylogenetic tree for PeiR PMBR1 and PMBR2 domains. Sequences used in this tree consist of the Pfam seed sequences for the PMBR family. RAxML was used for rapid bootstrapping and a subsequent Maximum Likelihood search using 1 distinct model/data partition with joint branch length optimisation. 100 rapid bootstrap inferences were executed and thereafter a thorough ML search calculated. All free model parameters have been estimated by RAxML. ML was estimated at 25 per site rate categories. The likelihood of final tree was evaluated and optimised under GAMMA. GAMMA model parameters have been estimated up to an accuracy of 0.1 Log Likelihood units. RAxML was called as follows: raxmlHPC -f a -x 12345 -p 12345 -# 100 -m PROTCATBLOSUM62 -s PMBR_length adjusted.phy -n PMBR with the following parameters: Partition: 0. Alignment Patterns: 36. Substitution Matrix: BLOSUM62. Using fixed base frequencies.


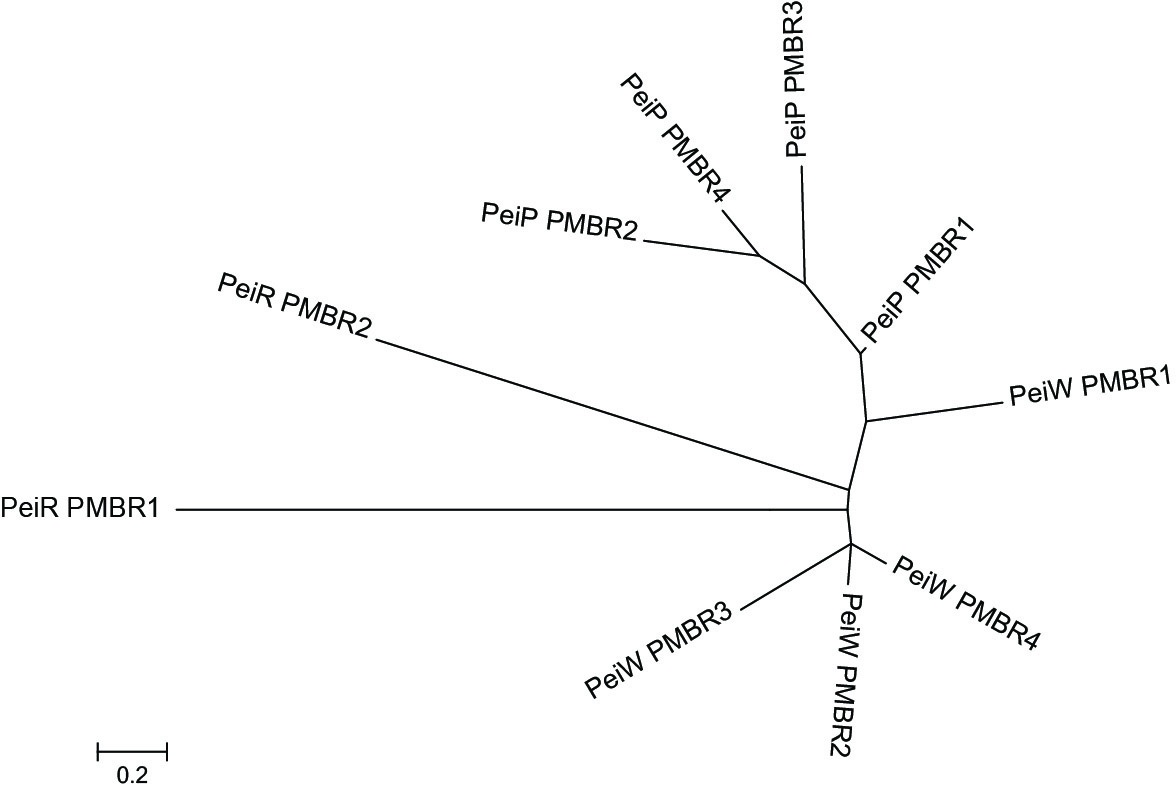


Supplemental Figure 3: Phylogenetic tree investigating the relationships between PMBR domains of PeiR, PeiP and PeiW. The evolutionary history was inferred by using the Maximum Likelihood method based on the JTT matrix-based model ([Jones et al., 1992](#_ENREF_24)). The tree with the highest log likelihood (-564.9445) is shown. Initial tree(s) for the heuristic search were obtained automatically by applying Neighbor-Join and BioNJ algorithms to a matrix of pairwise distances estimated using a JTT model, and then selecting the topology with superior log likelihood value. The tree is drawn to scale, with branch lengths measured in the number of substitutions per site. All positions containing gaps and missing data were eliminated. There were a total of 30 positions in the final dataset. Evolutionary analyses were conducted in MEGA5 (<http://www.megasoftware.net/>).


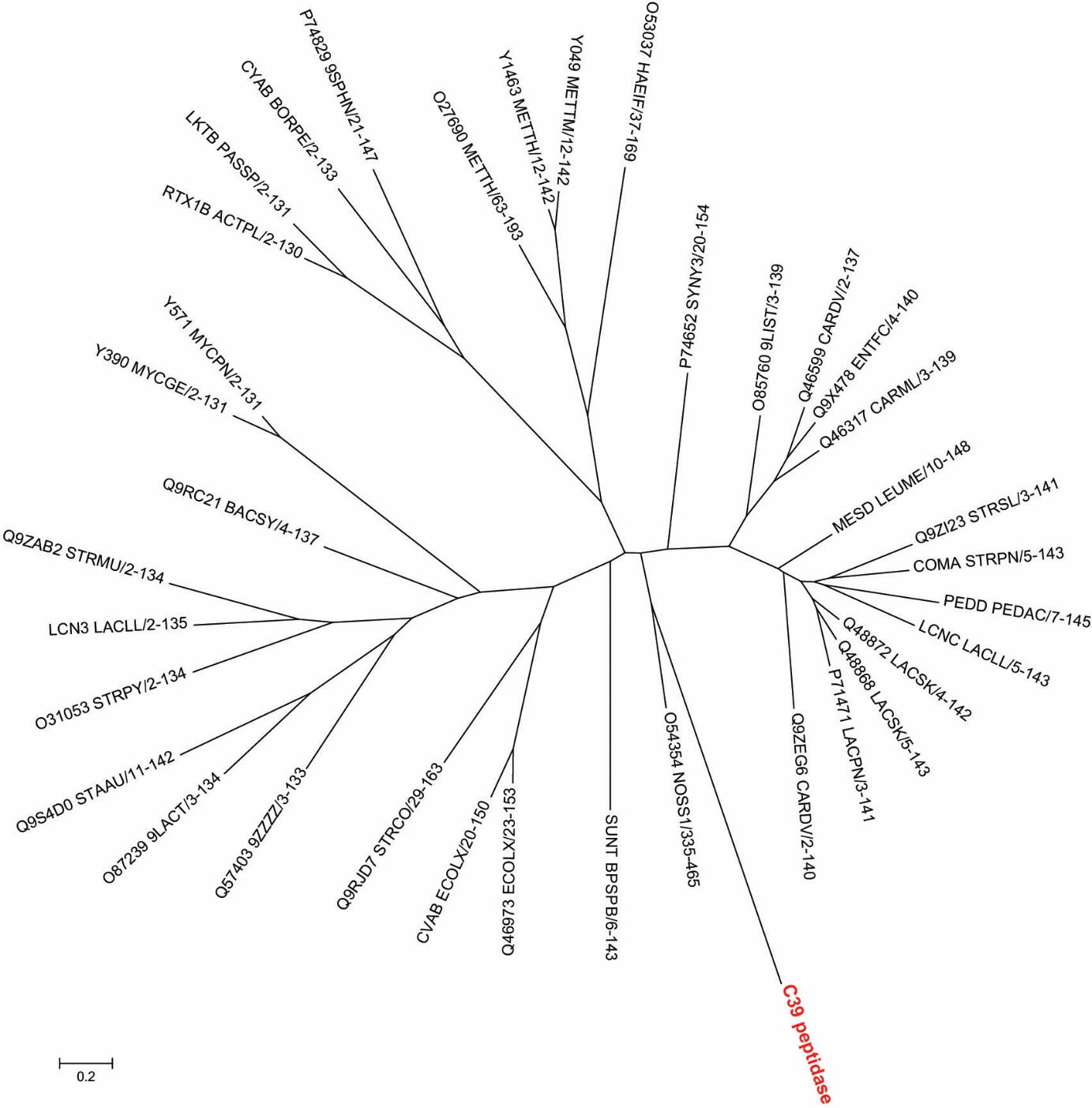


Supplemental Figure 4: Phylogenetic tree for PeiR C39 peptidase domain. Sequences used in this tree consist of the respective Pfam seed sequences for the C39 family. RAxML was used for rapid bootstrapping and subsequent ML search, using 1 distinct model/data partition with joint branch length optimisation. 100 rapid bootstrap inferences were executed and thereafter a thorough Maximum Likelihood (ML) search calculated. All free model parameters have been estimated by RAxML. ML was estimated at 25 per site rate categories. The likelihood of final tree was evaluated and optimised under GAMMA. GAMMA model parameters have been estimated up to an accuracy of 0.1 Log Likelihood units. RAxML was called as follows: raxmlHPC -f a -x 12345 -p 12345 -# 100 -m PROTCATBLOSUM62 -s C39_seed_alignment.phy -n C39_peptidase with the following parameters: Partition: 0. Alignment Patterns: 168. Substitution Matrix: BLOSUM62. Using fixed base frequencies.


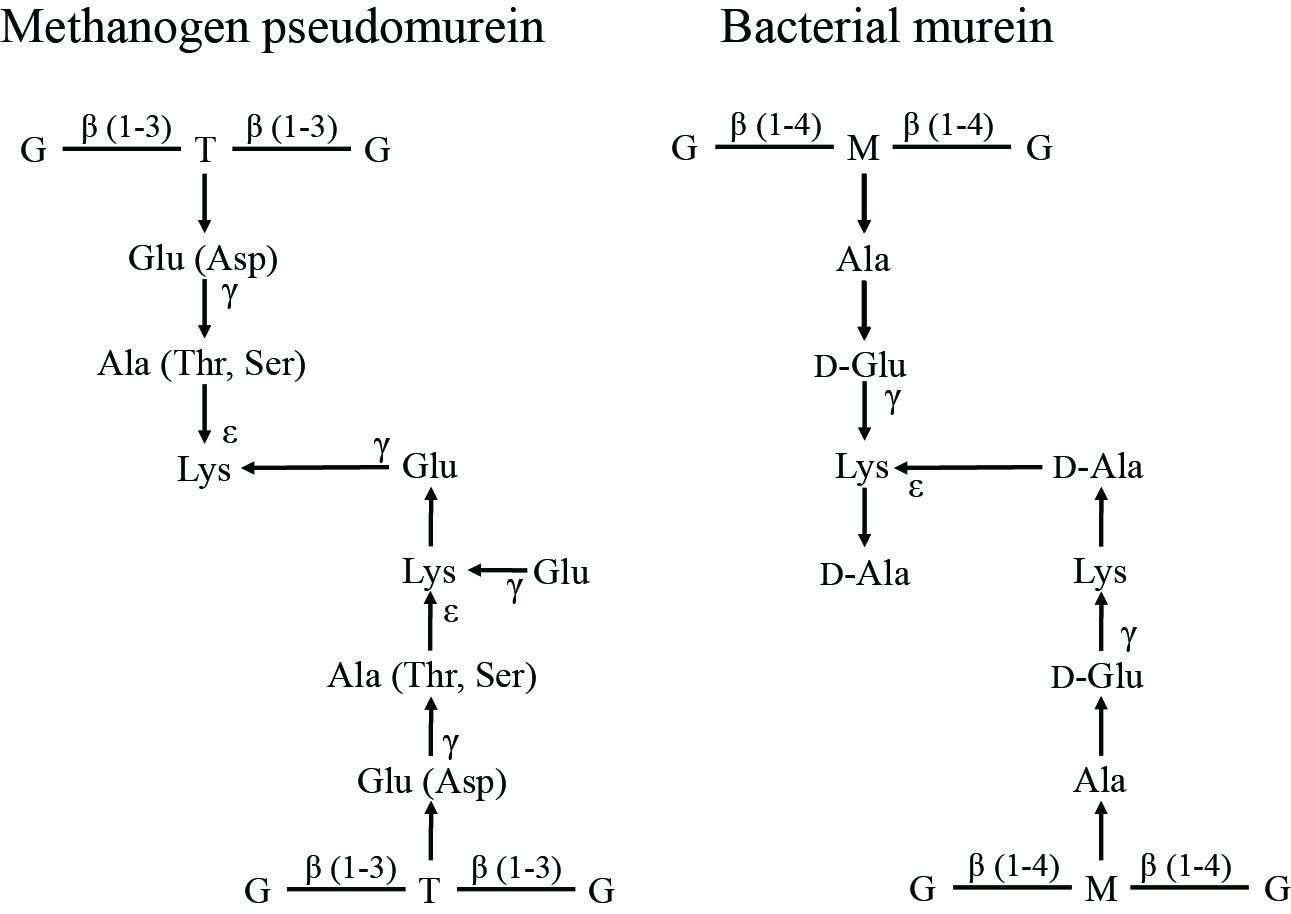


Supplemental Figure 5: Comparison of the composition of pseudomurein and murein from cell walls. G, *N*-acetylglucosamine; T, *N*-acetyltalosaminuronic acid; M *N*-acetylmuramic acid. Methanogen pseudomurein contains; L-amino acids, β (1-3) bonds and *N*-acetyltalosaminuronic acid in the glycan chain, and several unusual isopeptide bonds. Glu can be replaced by Asp; Ala can be replaced by Thr or Ser, in some strains. Bacterial murein contains; D- and L-amino acids, and β (1-4) bonds and *N*-acetylmuramic acid in the glycan chain. The figure was published previously ([Schofield et al., 2015](#_ENREF_52)) and was adapted and modified from Hartmann and König ([Hartmann and Konig, 1990](#_ENREF_19)), and Kandler and König ([Kandler and König, 1993](#_ENREF_25)).


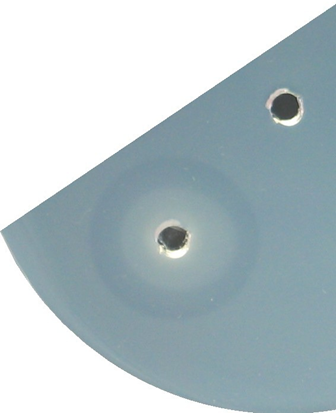


Supplemental Figure 6: Example of activity of free PeiR enzyme on methanogen cells as substrate in an agarose plate lysate assay. Plate contained *Methanothermobacter thermautotrophicus* ΔH cells. Lower well contained PeiR free enzyme, the diameter of the clear zone is 21 mm. Upper well contained buffer (control). The diameter of the wells is 4 mm.


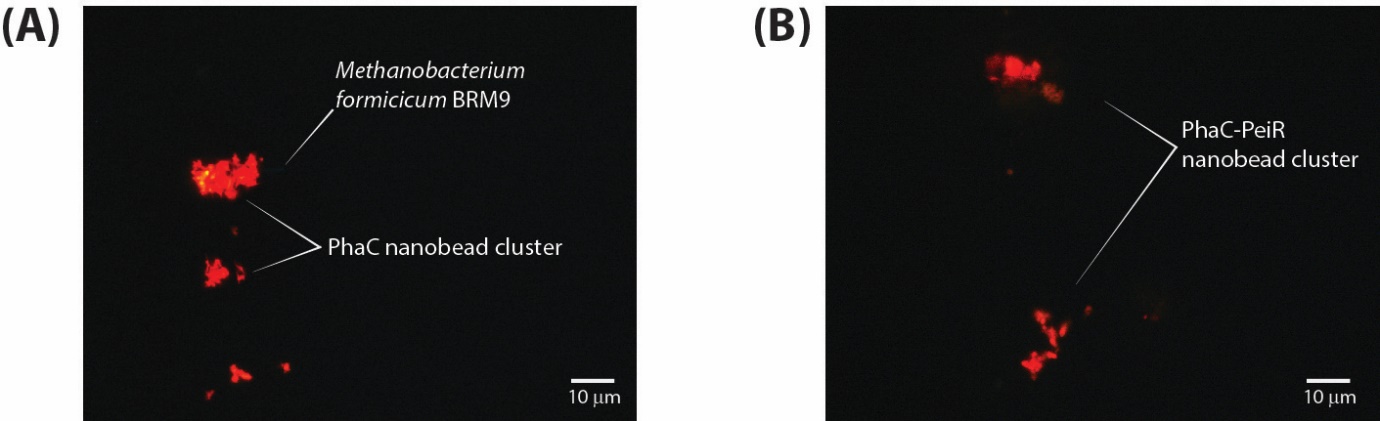


Supplemental Figure 7: Effect of BRM9 on (A) PhaC and (B) PhaC-PeiR BNPs. BNPs form large clusters that are not associated with individual cells, although many clusters take on shapes resembling that of BRM9 cells, albeit larger in diameter. This effect appeared to be unique to BRM9; no other tested methanogen strain resulted in aggregate formation of PhaC BNPs. BRM9 cells are shown in blue and Nile Red-loaded PhaC BNPs appear in orange. The auto-fluorescence of BRM9 cells appears very faint due to the strong fluorescence of Nile Red stained BNPs.

**Supplemental Text**

Supplemental Text 1: Detailed Analysis of PhaC-PeiR activity against rumen methanogens

Methanobrevibacter sp. 229/11 was isolated from a lamb rumen in New Zealand ([Kelly et al., 2016a](#_ENREF_27)). Based on 16S rRNA gene sequence analysis, Methanobrevibacter olleyae was found to be the closest type strain. Strain 229/11 falls into the same clade as M1, albeit forming a separate cluster. 229/11 features a pseudomurein containing cell wall, similar to that of M1. The addition of PhaC control BNPs to a growing culture of strain 229/11 led to a marked increase in optical density above the level observed for growth of 229/11 alone. Significant differences in optical densities between 229/11 and 229/11 plus PhaC cultures were found for days 3 and 4 (P<0.05), with 229/11 plus PhaC reaching a final optical density twice as high as 229/11 alone. In contrast, the presence of PhaC-PeiR BNPs caused a marked drop in culture optical density that remained stable over 72 hours until the end of the assay (Figure 5 A). Significant differences in optical densities between 229/11 plus PhaC-PeiR beads and 229/11 or 229/11 plus PhaC and were found at days 2, 3, and 4 (P<0.05). In parallel, the production of methane by 229/11 was measured over the course of four days. Methane production reached detectable quantities in the 229/11 control 48 hours after inoculation and then dropped to 0.148 and 0.17 ml (64% and 58% reduction from day 2) of methane on days 3 and 4, respectively. Although the optical densities of 229/11 plus PhaC increased steadily compared to 229/11, methane production remained at the same level over the 3 days where methane was detected (P=0.421). Treatment of 229/11 with PhaC-PeiR BNPs led to a significant reduction in methane (P<0.05). On day 2, methane production was reduced by 97% and 95% compared to control and PhaC treatment. Over the next 48 hours, methane production gradually increased to 0.078 ml, which represents a reduction of 57% and 73% for control and PhaC treatment, respectively. Both the drop in optical density and the reduction of methane are comparable to the effects seen in M1, indicating that PhaC-PeiR BNPs are similarly effective against both methanogen strains.

The New Zealand isolate *Methanobrevibacter* sp. AbM4 originated from a sheep abomasum and 16S rRNA sequence analysis found the closest type strain to be *Methanobrevibacter wolinii* *(*[*Leahy et al., 2013*](#_ENREF_37)*)*. Similar to strains M1 and 229/11, AbM4 falls within the genus *Methanobrevibacter* clade and features a pseudomurein cell wall. The rate of cell growth, as measured by optical density, reached a maximum 48 hours post-inoculation for both AbM4 and AbM4 plus PhaC cultures (Figure 5 B). Methane production mirrored this trend and the highest level of methane was found 48 hours post-inoculation (Figure 6 B). Optical densities of AbM4 and AbM4 plus PhaC cultures continued to increase on days 3 and 4 post inoculation, albeit at a lower rate. Correspondingly, methane production dropped by 50 to 70% to 0.18 ml and 0.11 ml for AbM4 and AbM4 plus PhaC cultures, respectively. The addition of PhaC-PeiR BNPs inhibited growth entirely for up to 48 hours post-inoculation (P<0.05). No methane was detected 24 hours after BNP addition, whereas a small amount (0.02 ml) was observed after 48 hours. Beyond 48 hours, PhaC-PeiR BNPs lose their ability to inhibit cell growth and after 4 days AbM4/PhaC-PeiR cultures reached optical densities similar to those found for AbM4 and AbM4 plus PhaC after 48 hours. Likewise, methane production increased and mirrored levels found for uninhibited cultures. Interestingly, an increase in optical density comparable to that seen in M1 plus PhaC and 229/11 plus PhaC did not follow the addition of PhaC BNPs, although on days 1 and 3 AbM4 plus PhaC cultures resulted in a statistically significant increase in optical density (P<0.05) over AbM4 alone.

The final *Methanobrevibacterium* tested, strain D5, was isolated from a New Zealand sheep rumen and 16S rRNA gene sequence analysis suggested it may fall into the *M. gottschalkii* clade with a pseudomurein cell wall. Similar to strains 229/11 and AbM4, the addition of PhaC BNPs did not result in any negative growth effects when compared to an unsupplemented culture of D5 (Figure 5 C). No statistically significant differences in optical densities were found between both cultures. Methane production followed the trend observed for strains 229/11 and AbM4 and peaked after 48 hours post inoculation, with no statistically significant differences between D5 and D5 plus PhaC cultures (Figure 6 C). D5 cultures supplemented with PhaC-PeiR BNPs were effectively inhibited in optical density for up to 3 days post inoculation, while on day 4 cell growth resumed (P<0.05). Accordingly, methane production was effectively inhibited for up to 48 hours post-inoculation (P<0.05), although small amounts of methane were detected on both days. Although optical densities indicate effective inhibition beyond 48 hours, methane levels on days 3 and 4 are equal to D5 and D5 plus PhaC cultures (no statistically significant differences between the treatments), possibly indicating that D5 cells may be less susceptible to the lytic enzyme PeiR.

In summary, tested *Methanobrevibacter* strains assigned to *M.* *ruminantium*, *M.* *olleyae*, *M.* *wolinii*, and *M.* *gottschalkii* can be effectively inhibited in cell growth and methane production for up to 48 to 72 hours using PhaC-PeiR tailored BNPs.

I*n vitro* assays suggested that free PeiR enzyme may be able to act on methanogen strains other than *Methanobrevibacter* spp. The activity of PhaC-PeiR BNPs against pseudomurein-containing methanogens outside the genus *Methanobrevibacter* was tested on two selected strains of *Methanobacterium* and *Methanosphaera*.

*Methanosphaera* sp. A4 was isolated from a New Zealand Wallaby fore stomach and 16S rRNA gene analysis revealed *Methanosphaera cuniculi* as the closest relative. As seen for *Methanobrevibacter* spp., the addition of PhaC BNPs to strain A4 led to an increase in measured optical density over the untreated culture, an effect seen for several other methanogen strains tested (Figure 5 D). The addition of PhaC-PeiR BNPs caused a statistically significant reduction in optical density on day 3 post-inoculation (P<0.05), similar to that reported for *Methanobrevibacter* spp. On days 4 and 5 PhaC-PeiR mediated inhibition was overcome with optical densities reaching similar levels to untreated and PhaC-treated cultures. Following this trend, methane production was significantly inhibited on days 2 and 3 post inoculation (P<0.05), whereas on day 4 measured methane levels were equal to the other two treatment groups. Methane production was not affected by the presence of PhaC BNPs.

*Methanobacterium formicicum* BRM9 is a New Zealand bovine rumen isolate and grows as narrow straight or slightly curved rods. Because of the aggregate forming interaction between PhaC/PhaC-PeiR BNPs and BRM9 cells, optical density could not be measured. The detection of methane, however, was not impaired and a statistically significant reduction of methane was detected on day 4 post inoculation of PhaC-PeiR BNPs when compared to untreated cells (P<0.05) (Figure 6 E).

Strain *Methanosarcina* *barkeri* CM1 was isolated from a bovine rumen in New Zealand and grows in irregular cell aggregates typical for *Methanosarcina*. The observed growth in aggregates prevented direct measurement of optical densities of liquid cultures. Although very weak activity of the PeiR free enzyme was seen against CM1 cell wall preparations, no reduction in methane was measured in pure cultures (Figure 6 F) which supports the hypothesis that PeiR can readily hydrolyse pseudomurein-based substrates, but not effectively recognise methanochondroitin-based cell envelopes.

Supplemental Text 2: Interpretation of observed data on the biological mode-of-action of PeiR

Methanogen cultures were able to recover after some time despite the presence of PhaC-PeiR BNPs. Similar to PeiR, the lytic enzymes PeiP and PeiW harbour PMBR domains that mediate binding to the pseudomurein cell wall. Those PMBR domains have been described to bind both highly specifically and strongly to pseudomurein ligands. *In vivo*, lytic enzymes act on the host cell only through a lysis-from-within mechanism. Once the host cell has lysed, the virus particles are liberated and are ready to infect a new cell, repeating the propagation cycle. Repeated binding-and-release of the lytic enzyme is, therefore, not required – rather biological activity may be optimised by the observed strong substrate binding. It is possible that this mechanism equally mediates the effective binding of PhaC-PeiR BNPs to compatible methanogen cells, facilitating a lysis-from-without. It is tempting to hypothesise that upon cell lysis, the pseudomurein substrate may remain bound to the PMBR domains of individual PeiR enzymes, slowly inactivating more and more enzymes until the cell-to-active enzyme ratio tips in favour of the methanogen cells.
